# Children Born to HIV-Infected Mothers in Côte d'Ivoire: Methodological Clarifications Needed

**DOI:** 10.1371/journal.pmed.0040140

**Published:** 2007-03-27

**Authors:** Moussa Sarr

In their recently published paper, Becquet et al. [1] found that the 2-years rates of adverse health outcomes were similar among short-term breast-fed and formula-fed children. Mortality rates also did not differ significantly between these two groups and, after adjustment for pediatric HIV status, were similar to those observed among long-term breast-fed children. These results confirm the findings of two previous trials in Kenya [2] and in Botswana [3], highlighting the fact that with adequate support, alternatives to prolonged breast-feeding can be safe options for mothers to prevent mother-to-child transmission of HIV in African settings. HIV-infected mothers who opt for alternatives to breast-feeding to protect their children from HIV infection should be provided the necessary support to make their choice feasible.

There are, however, some methodological clarifications that need to be made regarding the incidence rates of diarrhea, acute respiratory infection, and malnutrition. It was not clear if all repeated episodes of diarrhea and acute respiratory infection were taken into account to compute the incidence rates. A number of epidemiologists have also been advocating the use of longitudinal prevalence instead of incidence for the longitudinal measure of morbidity associated with childhood diarrhea [4]. The longitudinal prevalence is defined by the number of days of diarrhea divided by the total number of days of observation for each child. Longitudinal prevalence was found to be a better predictor of long-term health outcome in relationship to childhood diarrhea [4].
